# A New Root-Knot Nematode species, *Meloidogyne karsseni* n. sp. (Nematoda: Meloidogynidae), From Mexico and a Taxonomic Update on *M. paranaensis* From Guatemala

**DOI:** 10.2478/jofnem-2023-0042

**Published:** 2023-10-21

**Authors:** Phougeishangbam Rolish Singh, Denis Gitonga, Abolfazl Hajihassani, Adriaan Verhage, Eveline van Aalst-Philipse, Marjolein Couvreur, Wim Bert

**Affiliations:** Department of Entomology and Nematology, Fort Lauderdale Research and Education Center, Institute of Food and Agricultural Sciences, University of Florida; Davie, FL 33314, USA; Nematology Research Unit, Department of Biology, Ghent University, K.L. Ledeganckstraat 35, 9000 Ghent, Belgium; Rijk Zwaan Breeding B.V., Burgemeester Crezéelaan 40, PO Box: 40, 2678 ZG De Lier, the Netherlands

**Keywords:** Guatemala, *Meloidogyne*, Mexico, new species, phylogeny, Root-knot nematode, taxonomy

## Abstract

A new root-knot nematode (RKN) species, *Meloidogyne karsseni* n. sp., associated with sweet pepper from Mexico, and a population of *M. paranaensis* from Guatemala, are described using data from morphological, biochemical (isozyme enzymes), molecular, and phylogenetic analyses. *Meloidogyne karsseni* n. sp. can be morphologically diagnosed using the combined features of the second-stage juveniles, viz. body length (345 to 422 μm), a conical rounded head region, a post-labial annule lacking transverse striation, a thin stylet 11 to 12 μm long, rounded to oval and backwardly sloping knobs, dorsal gland orifice (DGO) at 5.2 to 6.0 μm from the knobs, a hemizonid just above the secretory-excretory (SE) pore, a tapering tail with finely rounded terminus and one or two very weak constrictions at hyaline tail tip; the female characters viz. oval-to-rounded perineal pattern with coarse striation on lateral sides around the anus, low dorsal arch with finer striations, and distinctly visible lateral lines; and the male characteristics viz. a rounded and continuous head, a post-labial annule without transverse striations, a robust stylet 20 to 24 μm long, rounded-to-oval and slightly backwardly sloping knobs, and a DGO at 2.4 to 2.9 μm from the knobs. In all the studied males of *M. paranaensis*, a characteristic sclerotization around the duct of SE-pore was also observed for the first time. Sequences of 18S, D2–D3 of 28S, and ITS of rDNA, and *cox*1 of mtDNA were generated for the two species, and in the phylogenetic trees based on these genes, both species appeared in the tropical RKN species complex clade.

Members of the plant-parasitic nematode (PPN) genus *Meloidogyne* Göldi, 1887 (Nematoda: Meloidogynidae) are commonly known as root-knot nematodes (RKN) because of their ability to induce knots on the root systems of host plants, resulting in significant disorder in the physiology of the infected plants (Perry et al., 2009). Ranked as the most scientifically and economically important PPN group (Jones et al., 2013), many RKN species have been listed as notorious parasites of a wide range of agricultural crops (Karssen, 2002; Bridge and Starr, 2007; Perry et al., 2009; Sikora et al., 2018), and several species have also been listed as quarantine agricultural pests in different parts of the world (Knoetze et al., 2017; EPPO 2022; Subbotin et al., 2021). The RKN are obligate root-endoparasites with second-stage juveniles (J2) that migrate within soil, invading host root tips and move intra- and intercellularly inside the root. A susceptible plant reacts to the J2 feeding, usually by forming giant cells in the roots, where the J2 become sedentary and feed from these cells. The J2 subsequently molt to become adults, whereupon the females continue feeding, become saccate and start laying eggs into egg sacs (Perry et al., 2009). The most prominent symptom of RKN infection is galling of the host's roots, with the severity of the galling ranging from none to massive galls all over the root systems, thereby causing serious root deformations (Bridge and Starr, 2007; Coyne, 2007).

In the most recent taxonomic update of *Meloidogyne* by Subbotin et al. (2021), 98 nominal species have been listed, along with seven *species inquerendae* and six *nomina nuda*. The diagnosis of these PPN is generally carried out using an integrated approach, based on morphological, molecular, and biochemical analyses. Generally, RKN species can be morphologically diagnosed using the J2 characteristics such as lip region, stylet length, length of dorsal gland orifice (DGO) from the knobs, position of hemizonid and secretory-excretory (SE) pore, tail and tail hyaline lengths, and tail tip shape. For the adult stages, female characteristics, such as stylet length and perineal pattern, and male characteristics, such as lip region, stylet length, knob shape and the DGO length from the knobs (Jepson, 1987; Karssen, 2002), can be used. Additionally, sequence variations of ribosomal and mitochondrial gene fragments (D2–D3 of 28S, partial 18S, ITS, *cox* I–III, *nad*5, etc.) and isozyme patterns (esterase and malate dehydrogenase isozyme phenotypes) can also be used as taxonomic markers for the identification of *Meloidogyne* species (Karssen, 2002; Subbotin et al., 2021; Perry et al., 2020).

This current work studied a new *Meloidogyne* species associated with sweet pepper (*Capsicum anuum* L.) from Mexico and a population of *M. paranaensis* Carneiro, Carneiro, Abrantes, Santos & Almeida, 1996 that originated from a greenhouse culture at the National Plant Protection Organization (NPPO), Wageningen, the Netherlands. Morphological, molecular, and biochemical data were generated for the two species; the morphological characterization included light and scanning electron microscopy images, line illustrations, and morphometrics, while the biochemical characterization was based on esterase and malate dehydrogenase isozyme patterns. The molecular and phylogenetic analyses were based on the D2–D3 region of 28S, partial 18S, and the ITS of rDNA, and *cox*1 of mtDNA sequences.

## Materials and Methods

### Nematode collection and extraction

The new species was found to be associated with sweet pepper in 2019 in Mexico and has been maintained in the greenhouse of the NPPO in potted tomato (*Solanum lycopersicum* L.) plants (NPPO collection number: F5919). The *M. paranaensis* population was received by the NPPO from Guatemala in 1995 as a RKN-infested tomato root sample. This population has also since been in the greenhouse culture at the NPPO in potted tomato plants (NPPO collection number: C7729).

The J2 and males of both species were extracted from about 100 ml of soil from their respective culture pots by the centrifugation method (van Bezooijen, 2006). Obese females were directly picked out from the root galls of the host plants using fine forceps, needles, and glass pipettes. The nematodes were stored at 4°C during the study.

### Morphological characterization

Both temporary mounts of heat-killed and permanent mounts of chemically fixed nematode specimens were used for morphological characterization. For preparation of a temporary mount of a J2 or male, a Cryo-Pro label (VWR International) was cut into two halves and stuck at the center of a glass slide, leaving a small parallel gap between them. A single specimen was transferred in a drop of distilled water onto the glass slide in the center of the gap, then the slide was passed intermittently over a flame until nematode movements stopped. The specimen was covered with a glass coverslip and examined, photographed, and measured using an Olympus BX50 DIC Microscope (Olympus Optical, Tokyo, Japan), equipped with a UCMOS 5MP camera. After recording morphological data, individual specimens were recovered from the slide by adding a few drops of water from one end of the gap and collecting the nematodes that were flushed out from the other end of the gap (Singh et al., 2021). Obese female specimens were individually teased out from galled roots in water in a petri dish using two fine needles and pipetted into a new embryo glass dish. The recovered specimens were subsequently used to extract genomic DNA, as described in this manuscript. For making permanent mounts, live nematodes were killed and fixed using warm 4% formaldehyde (about 60°C) and left in the fixative for 10 days at 4°C. The fixed nematodes were gradually transferred to glycerin (Seinhorst, 1959). The fixed specimens were mounted in glycerin on glass slides, covered with glass coverslips and studied using the camera-equipped compound microscope. Drawings were made with the aid of a drawing tube attached to the microscope and further improved using Photoshop CS6 (Adobe, San Jose, CA).

For scanning electron microscopy (SEM), specimens fixed in Trump's fixative (2% paraformaldehyde, 2.5% glutaraldehyde in 0.1 M Sorenson buffer [sodium phosphate buffer at pH = 7.3]) were washed in 0.1 M phosphate buffer (pH = 7.5) and dehydrated in a graded series of ethanol solutions, critical point-dried with liquid CO_2_, mounted on stubs with carbon tabs (double conductive tapes), coated with 25 nm of gold, and photographed with a JSM-840 EM (JEOL, Tokyo, Japan) operating at 12 kV (Singh et al., 2020).

### Molecular characterization

Individual nematodes (J2 and females) were used to extract genomic DNA. The cuticle of each nematode was first punctured with the help of a metallic pin used as a worm-picking tool, and transferred to a polymerase chain reaction (PCR) tube containing 20 μl of worm lysis buffer (50 mM KCl, 10 mM Tris at pH = 8.3, 2.5 mM MgCl_2_, 0.45% NP 40 [Tergitol Sigma Belgium], and 0.45% Tween 20). The PCR tubes were then frozen at −20°C (10 min), then 1 μl proteinase K (1.2 mg/ml) was added. The mixture was incubated at 65°C (1 h) and 95°C (10 min), and finally the lysate was centrifuged at 14,000 × g for 1 min (Singh et al., 2020).

The D2–D3 of 28S, partial 18S and ITS of rDNA were amplified by PCR using the primer pairs D2A: 5′-ACA AGT ACC GTG AGG GAA AGT TG-3′/D3B: 5′-TCC TCG GAA GGA ACC AGC TAC TA-3′ (Nunn, 1992), SSU18A: 5′-AAA GAT TAA GCC ATG CAT G-3′/SSU26R: 5′-CAT TCT TGG CAA ATG CTT TCG-3′ (Mayer et al., 2007) and Vrain2F: 5′-CTT TGT ACA CAC CGC CCG TCG CT-3′/Vrain2R: 5′-TTT CACT CGC CGT TAC TAA GGG AAT C-3′ (Vrain et al., 1992) respectively, and following the thermal profiles described in Singh et al. (2019). For amplification of the partial sequence of the mitochondrial *cox*1 gene, the primer pair JB3: 5′-TTT TTT GGG CAT CCT GAG GTT TAT-3′/JB4.5: 5′-TAA AGA AAG AAC ATA ATG AAA ATG-3′ (Bowles et al., 1992) was used with thermal profile from Etongwe et al. (2020). The PCR products were enzymatically cleaned using alkaline phosphatase (1 U/ml) and exonuclease I (20 U/ml) for 15 min at 37°C followed by 15 min at 80°C (Singh et al., 2020), and sequenced at Macrogen (Seoul, South Korea, https://dna.macrogen.com). The contigs were made from the newly produced forward and backward sequences using Geneious Prime 2020.0.5 (Dotmatics, Boston, MA, https://www.geneious.com) and were deposited in GenBank.

The isozyme characterizations were based on esterase (Est; EC 3.1.1.1) and malate dehydrogenase (Mdh; EC 1.1.1.37) staining of individual young egg-laying females (Karssen et al., 1995).

### Phylogenetic analysis

The phylogenetic relationships of the two species with other RKN species were analyzed based on the D2–D3, ITS, 18S, and *cox1* sequences. Phylogenetic programs implemented in Geneious Prime 2020.0.5 were used. The obtained consensus contigs were subjected to BLAST search to check for closely related species in GenBank. All of the collected sequences for each gene fragment were aligned using MUSCLE alignment in Geneious Prime 2020.0.5 using default parameters, followed by manual trimming of the poorly aligned ends. Bayesian phylogenetic analysis (MrBayes 3.2.6) was carried out using the GTR + I + G nucleotide substitution model, analyses were run under 1 × 10^6^ generations (4 runs), with Markov chains being sampled every 200 generations. 20% of the converged runs were regarded as burn-in (Huelsenbeck and Ronquist, 2001).

## Results and Description

### *Meloidogyne karsseni* n. sp.

**Figure 1:**
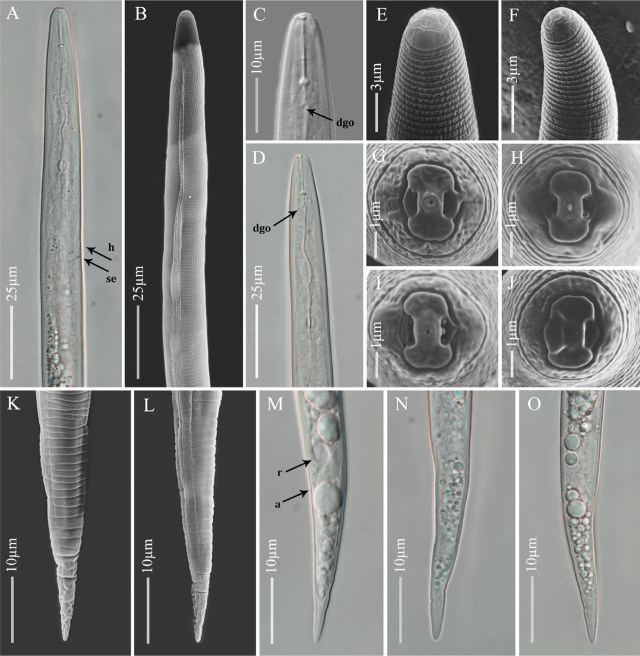
Light and scanning electron microscopy images of *Meloidogyne karsseni* n. sp. second-stage juveniles (J2). A, B: Anterior body up to pharyngeal gland end showing hemizonid (*h*) and secretory-excretory pore (*se*); C–F: Anterior end showing lip region, stylet and dorsal gland orifice (*dgo*); G–J: *En face* views; K–O: Posterior body showing rectum (*r*), anus (*a*), tail and hyaline part of tail.

**Figure 2:**
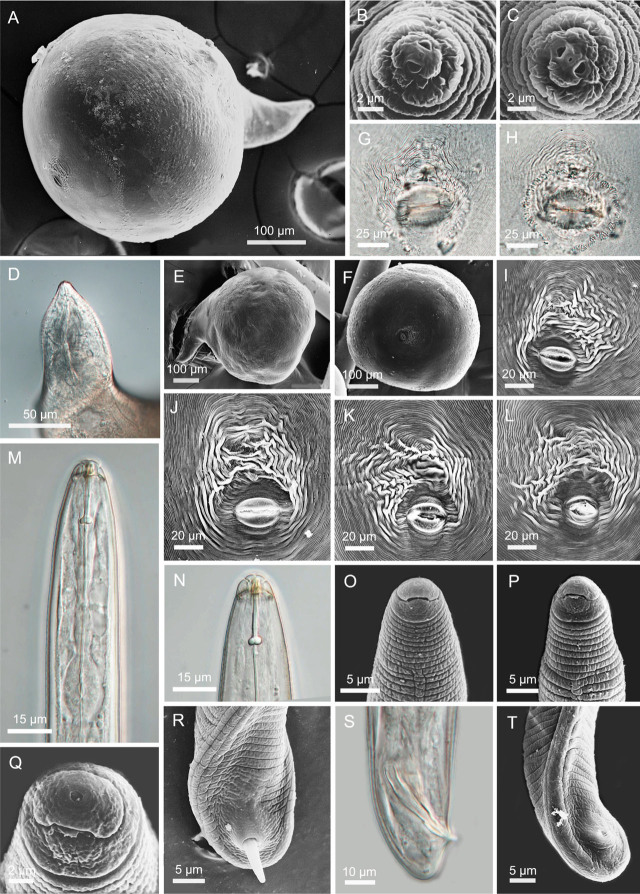
Light and scanning electron microscopy images of *Meloidogyne karsseni* n. sp. A, E: Whole body of female; B, C: *En face* of females; D: Neck region of female showing stylet and bulb; F–L: Female perineal patterns (G: outside view of cuticle; H: view from inside of cuticle); M–P: Anterior body of male showing lip region, stylet and dorsal gland orifice; Q: *En face* view of male; R–T: Posterior body of male showing spicules.

**Figure 3:**
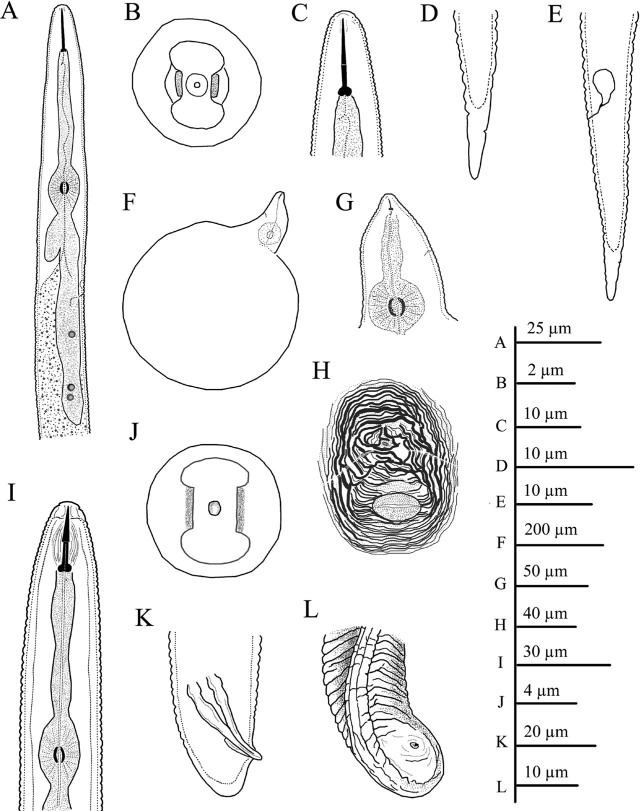
Line illustrations of *Meloidogyne karsseni* n. sp. A: Anterior body of second-stage juveniles (J2) showing up to gland end; B: *En face* view of J2; C: Anterior end of J2 showing stylet and dorsal gland orifice; D, E: Posterior end of J2; F: Whole body of female; G: Neck region of female; H: Female perineal pattern; I: Anterior body of male; J: *En face* view of male; K, L: Posterior body of male.

Figures 1–3, Table 1.

**Table 1.** Morphometrics of *Meloidogyne karsseni* n. sp. from fixed specimens mounted in glycerin. Measurements are given in μm and presented as average ± standard deviation (minimum–maximum).

	**Holotype female**	**Females**	**Juveniles**	**Males**
Total specimens	1	14	15	5
Body length (with neck in females)	878	821 ± 139 (630–1093)	395 ± 23.9 (345–422)	1463 ± 336 (1260–1850)
a	1.5	1.6 ± 0.2 (1.3–2.0)	32 ± 1.9 (27.6–34.1)	50 ± 7.8 (41.3–56.5)
c	-	-	8.7 ± 0.3 (8.4–9.2)	131 ± 24.9 (116–160)
c′	-	-	5.4 ± 0.4 (4.7–5.9)	0.5 ± 0.1 (0.5–0.6)
Stylet length	15.6	13.7 ± 1.3 (11.8–15.6)	11.8 ± 0.4 (10.8–12.4)	21.5 ± 1.7 (20.1–23.8)
DGO	3.8	3.3 ± 0.3 (2.9–3.8)	5.4 ± 0.3 (5.2–6.0)	2.7 ± 0.4 (2.4–2.9)
Knobs height	-	-	-	2.6 ± 0.3 (2.2–3.0)
Knobs width	-	-	-	4.5 ± 0.6 (3.7–5.4)
Lip height	-	-	-	5.1 ± 0.5 (4.6–5.6)
Lip width	-	-	-	10.0 ± 1.3 (8.6–11.4)
Anterior end to valve	87.0	85.9 ± 7.5 (76.7–103)	52.4 ± 1.5 (49.7–55.2)	86.7 ± 17.6 (70.1–113)
Median bulb length	61.0	43.4 ± 5.9 (38.2–61.0)	-	-
Median bulb width	54.0	41.7 ± 5.1 (34.9–54.0)	-	-
Anterior end to SE-pore	78.3	114 ± 25.7 (78.3–139)	78.9 ± 2.5 (73.7–82.6)	155 ± 22.6 (139–171)
Maximum body width	590	532 ± 87.4 (370–619)	12.3 ± 0.6 (11.0–13.3)	30.5 ± 12.4 (22.3–44.8)
Anal body width	-	-	8.6 ± 0.4 (7.7–9.3)	18.7 ± 1.3 (17.0–19.8)
Spicule length	-	-	-	27.7 ± 2.7 (25.0–30.0)
Gubernaculum length	-	-	-	7.8 ± 0.8 (7.2–8.4)
Tail length	-	-	46.1 ± 1.9 (43.7–49.1)	11.3 ± 0.4 (10.9–11.6)
Hyaline portion length	-	-	11.7 ± 1.0 (9.5–12.9)	-

### Description

#### Second-stage juveniles (J2)

Body vermiform, 0.3 to 0.4 mm long. Body annuli small and distinct. Lateral field differentiation starting at 24 to 25^th^ body annule as single band, developing into two bands, and eventually forming three bands, demarcated by four lines. Bands more or less of same width and irregularly areolated. Head region conical to rounded, continuous with body. Cephalic framework weakly sclerotized. As seen in SEM images, labial disc with central pore-like oral opening, fused with medial lips resulting in a well-marked dumbbell-shaped *en face* view; amphidial fovea on lateral sides. Single post-labial annule without transverse striations. Stylet thin and distinct, *ca* 12 μm long, knobs rounded-to-oval and backwardly sloping. DGO at 5.2 to 6.0 μm from knobs. Metacorpus distinct and ovoid. Gland lobe well developed, overlapping intestine ventrally. Hemizonid just above SE-pore. Rectum generally inflated. Tail tapers gradually to a finely rounded terminus. Tail hyaline part distinct, about 25% of tail length, usually with one or two very weak constrictions.

#### Females

Body large and globular with prominent neck, average length of 0.8 mm with neck and body width of 0.5 mm; clear annulation at neck region and pearly white in color. Stylet thin, 12 to 16 μm long, conus tip slightly curved dorsally; knobs rounded-to-transversely-ovoid and slightly sloping backward. SE-pore between stylet knobs and metacorpus level. Perineal pattern oval-to-rounded, striae coarse especially on lateral sides around anus, dorsal arch low with finer striations, lateral lines distinctly visible.

#### Males

Body large, 1.3 to 1.8 mm long, tapering anteriorly. Lateral field differentiation starting at 10 to 11^th^ annule as two lines forming a single band, developing into four lines forming three bands of equal width, outer bands areolated irregularly but mostly with complete areolations; mid band with sparsely scattered, mostly incomplete areolations. Head more or less continuous with body, rounded, with single post-labial annule without transverse striations. Labial disc curved, elevated and fused with medial lips. Stylet robust, 20 to 24 μm long; knobs rounded to oval, slightly backwardly sloping. DGO close to knobs. Hemizonid position variable, just before SE-pore in some specimens. Pharyngeal lumen usually well visible until valves of median bulb, pharyngeal gland overlapping intestine ventrally. Tail short and twisted. Spicules 25 to 30 μm long. Gubernaculum simple, slightly curved ventrally.

### Etymology

The species is named in honor of the eminent nematologist, Prof. Dr. Gerrit Karssen, for his valuable and exemplary contributions as a nematode taxonomist, especially in the RKN group.

### Type host

Sweet pepper (*Capsicum annuum* L.).

### Type locality

Guanojuato state in the city of San Jose Iturbide, Mexico.

### Type material

Holotype female and paratypes of females, J2 and males have been deposited at the Wageningen Nematode Collection (WaNeCo), Wageningen, the Netherlands. Additionally, paratypes of all life stages have also been deposited at Ghent University Zoological Museum and UGent Nematode Collection of the Nematology Research Unit of Ghent University, Belgium (slide: UGMD_104440).

### Morphological differential diagnosis and relationships with other *Meloidogyne* species

*Meloidogyne karsseni* n. sp. is characterized by J2 of 0.3 to 0.4 mm long with conical-rounded continuous head, post-labial annule without transverse striation, thin stylet of 11 to 12 μm, DGO at 5.2 to 6.0 μm from the knobs, hemizonid just above SE-pore, tail tapering to a finely rounded terminus and hyaline part bearing one or two very weak constrictions; globular females have rounded to oval perineal patterns with coarse striations, especially on the lateral sides around anus, and low dorsal arch with fine striations; males are without striations on the post-labial annule, with 20 to 24 μm long robust stylet and DGO at 2.4 to 2.9 μm from the knobs.

This species can be morphologically separated from all the known 20 species of the tropical RKN species complex, and especially from the five most common and also morphologically close RKN species found in North, Central, and South America – *M. arabicida* López & Salazar, 1989; *M. izalcoensis* Carneiro, Almeida, Gomes & Hernandez, 2005; *M. lopezi* Humphreys-Pereira, Flores-Chaves, Gomez, Salazar, Gomez-Alpízar & Elling, 2014; *M. konaensis* Eisenback, Bernard & Schmitt, 1994; and *M. paranaensis* (Subbotin et al., 2021).

*Meloidogyne karsseni* n. sp. is morphologically close to *M. arabicida* in that they have similar perineal patterns, stylet length in all stages, spicule length, ‘a’ and ‘c’ ratios in J2, tail, and tail hyaline lengths. However, it can be differentiated from *M. arabicida* based on the DGO lengths in males (2.4 to 2.9 vs. 3 to 5 μm from knobs) and in the J2 (5.2 to 6.0 vs. 2.0 to 4.7 μm), and absence vs. presence of transverse striations on the post-labial annule of both male and J2.

*Meloidogyne karsseni* n. sp. and *M. izalcoensis* are similar in J2, female and male stylet lengths; the absence of transverse striation on post-labial annule of both J2 and males; and ‘a’ and ‘c’ ratios, tail, and tail hyaline lengths of J2. However, the new species can be separated from *M. izalcoensis* based on the lengths of the DGO from knobs in females (2.9 to 3.8 vs 4.5 to 6.0 μm), males (2.4 to 2.9 vs. 4.0 to 7.0 μm), and the J2 (5.2 to 6.0 vs. 3.0 to 4.0 μm), and female perineal patterns (coarse striations around anus with low dorsal arch containing fine striations vs. coarse striations around the vulval lips and with a comparatively higher dorsal arch).

The new species and *M. lopezi* are similar in the J2 and male stylet length; male DGO length from knobs; absence of transverse striation on the post-labial annule of both the J2 and males; the ‘a’ and ‘c’ ratios; tail and hyaline lengths; and tail shape of the J2. However, *M. karsseni* n. sp. differs from *M. lopezi* by having a shorter female stylet (11.8 to 15.6 vs. 15 to 23 μm), more posterior DGO position in the J2 (5.2 to 6.0 vs. 2.5 to 3.5 μm), and female perineal pattern (lower dorsal arc with fine striations vs. comparatively higher dorsal arch with coarser striations).

*Meloidogyne karsseni* n. sp. and *M. konaensis* are similar in male stylet lengths; DGO position in J2; and lack of transverse striation on the post-labial annule in J2 and males. However, they differ in the stylet lengths of females (11.8 to 15.6 vs. 14.0 to 20.0 μm) and J2 (10.8 to 12.4 vs. 12.6 to 15.0 μm); DGO lengths in females (2.9 to 3.8 vs. 3.4 to 7.0 μm) and males (2.4 to 2.9 vs. 5 to 9 μm); absence vs. presence of numerous large projections on stylet shaft of male; and the lip region of the J2 (conical-rounded vs. more truncated).

*Meloidogyne karsseni* n. sp. is similar to *M. paranaensis* in J2 and male stylet lengths, J2 tail and hyaline lengths, and the ‘a’ and ‘c’ ratios. However, *M. karsseni* n. sp. differs from *M. paranaensis* by the absence vs. presence of transverse striation on the post-labial annule of both males and the J2; shorter female stylet length (11.8 to 15.6 vs. 15.0 to 17.5 μm); DGO length in the females (2.9 to 3.8 vs. 3 to 6 μm), males (2.4 to 2.9 vs 3.0 to 5.5 μm), and J2 (5.2 to 6.0 vs 2.0 to 4.5 μm); and the female perineal pattern (low dorsal arch vs. higher dorsal arch).

Lastly, the new species can be easily separated from the globally distributed tropical RKN species – *M. arenaria* (Neal, 1889) Chitwood, 1949, *M. incognita* (Kofoid & White, 1919) Chitwood, 1949, and *M. javanica* (Treub, 1885) Chitwood, 1949 – based on the combination of the following features: absence of transverse striation on post-labial annule and longer DGO length (>5 μm) in the J2; perineal patterns with coarse striations, especially on the lateral sides of anus; and very low dorsal arch.

### *Meloidogyne paranaensis* Carneiro, Carneiro, Abrantes, Santos & Almeida, 1996

**Figure 4:**
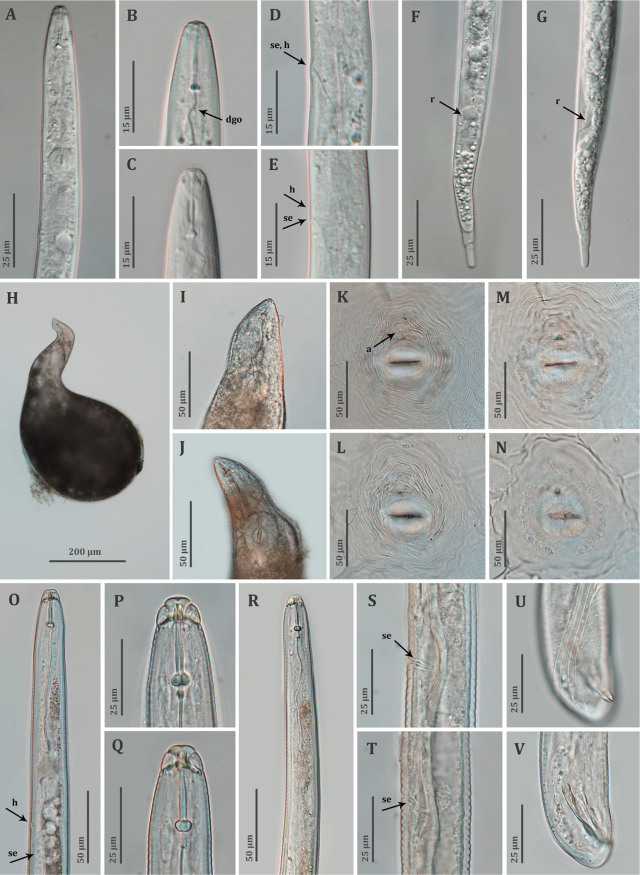
Light microscopy images of *Meloidogyne paranaensis*. A–C: Anterior body of second-stage juveniles (J2) showing lip region, stylet, knobs and dorsal gland orifice (*dgo*); D, E: Secretory-excretory (*se*) pore and hemizonid (*h*) of J2; F, G: Posterior body of J2 showing inflated rectum (*r*), tail and tip; H: Whole body of female; I, J: Anterior body of females showing stylet, knobs and mid-bulb of pharynx; K–N: Perineal patterns of females and anus (*a*) (K, L: outside view of cuticle; M, N: view from inside of cuticle); O–R: Anterior body of males showing lip region, stylet, knobs, dorsal gland orifice, secretory-excretory pore and hemizonid; S, T: Cuticular thickenings at secretory-excretory pore of males; U, V: Posterior body of male showing spicules and gubernaculum.

**Figure 5:**
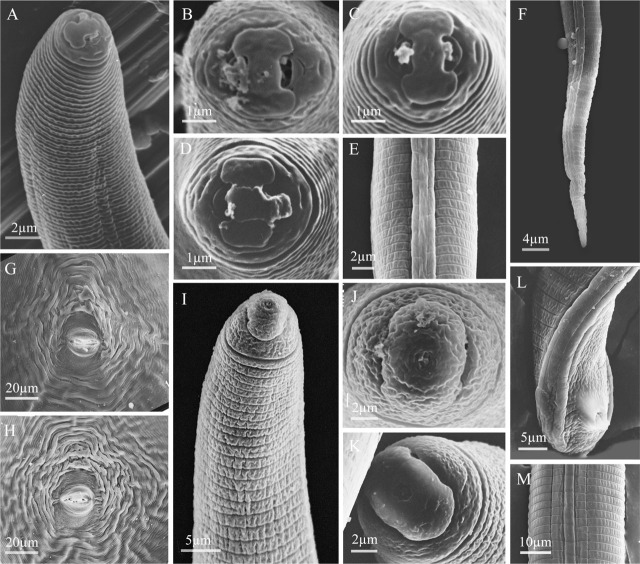
Scanning electron microscopy images of *Meloidogyne paranaensis*. A: Anterior body of second-stage juveniles (J2) showing lip region; B–D: *En face* views of J2 showing oral openings, labial disc, amphids, post-labial annule with incomplete transverse striations; E: Mid-body of J2 showing four lateral lines forming three bands; F: Posterior body of J2 showing tail and tip; G, H: Perineal patterns of females; I: Anterior body of a male showing lip region; J, K: *En face* view of males showing oral openings, labial disc, amphids and post-labial annule; L: Posterior body of male; M: Mid-body of a male showing four lateral lines forming three areolated bands.

**Figure 6:**
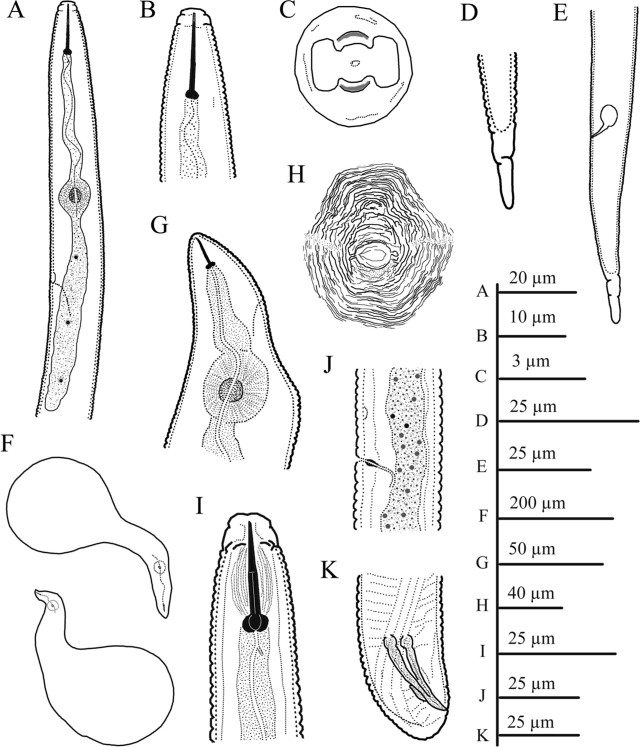
Line illustrations of *Meloidogyne paranaensis*. A, B: Anterior body of second-stage juveniles (J2); C: *En face* view of J2; D, E: Posterior body of J2; F: Whole female bodies; G: Anterior body of female; H: Perineal pattern of female; I: Anterior body of male; J: Cuticular thickenings of duct near secretory-excretory pore of male; K: Twisted tail of male showing spicules and gubernaculum.

Figures 4–6, Table 2.

**Table 2.** Morphometrics of *Meloidogyne paranaensis* Carneiro, Carneiro, Abrantes, Santos, & Almeida, 1996 from fixed specimens mounted in glycerin. Measurements are given in μm and presented as average ± standard deviation (minimum–maximum).

	**Females**	**Juveniles**	**Males**
Total specimens	10	25	10
Body length (with neck in females)	816 ± 91.4 (700–1005)	431 ± 14.3 (400–459)	1480 ± 232 (1012–1811)
a	1.9 ± 0.5 (1.3–3.1)	33.5 ± 1.4 (31.3–35.8)	49.0 ± 5.1 (40.2–55.1)
c	-	8.9 ± 0.4 (8.2–9.5)	122 ± 21.6 (92.4–156)
c′	-	5.5 ± 0.3 (4.7–6.0)	0.7 ± 0.1 (0.5–0.7)
Stylet length	17.7 ± 1.4 (16.2–20.1)	13.6 ± 0.4 (13.0–14.6)	23.2 ± 0.9 (21.6–24.7)
DGO	-	3.5–4.6	3.2 ± 0.3 (2.8–3.7)
Knobs height	-	-	2.9 ± 0.3 (2.6–3.5)
Knobs width	-	-	5.3 ± 0.6 (4.4–6.4)
Lip height	-	2.3 ± 0.2 (2.0–2.6)	6.2 ± 0.8 (5.2–7.3)
Lip width	-	4.4 ± 0.2 (4.1–4.8)	10.8 ± 0.9 (9.5–12.1)
Anterior end to valve length	87.0 ± 10.0 (70.0–99.0)	57.6 ± 1.7 (53.2–61.6)	89.6 ± 12.8 (66.0–107)
Median bulb length	39.7 ± 4.6 (29.2–44.5)	-	-
Median bulb width	37.9 ± 6.4 (24.4–44.3)	-	-
Anterior end to SE-pore	74.7 ± 22.6 (40.7–93.0)	82.4 ± 2.7 (77.2–85.9)	154 ± 14.8 (136–183)
Maximum body diameter	442 ± 70.7 (326–579)	12.8 ± 0.4 (12.1–13.7)	30.5 ± 5.7 (21.8–39.3)
Anal body diameter	-	8.9 ± 0.5 (8.0–10.1)	20.4 ± 1.4 (18.9–22.6)
Spicule length	-	-	34.0 ± 3.5 (30.4–38.7)
Gubernaculum length	-	-	9.4 ± 1.8 (7.9–11.9)
Tail length	-	48.7 ± 1.7 (45.7–52.0)	13.4 ± 1.3 (11.6–14.8)
Hyaline portion length	-	14.2 ± 0.9 (12.7–16.0)	-

### Description

#### Second-stage juveniles (J2)

Body vermiform, 0.4 to 0.5 mm long. Body annules small but distinct. Lateral field with four lines forming three bands of equal width and with irregular areolations of bands. Head region lowly rounded-to-truncated, more or less continuous with the rest of the body. Cephalic framework weakly sclerotized. *En face* view showing a dumbbell-shaped labial disc, well-marked, fused with medial lips, central pore-like oral opening, and amphidial fovea on lateral sides. Single post-labial annule with incomplete transverse striations. Stylet thin and distinct, *ca* 14 μm long; knobs transversely ovoid and slightly sloping posteriorly. DGO at 3.5 to 4.6 μm from knobs. Metacorpus distinct and ovoid. Gland lobe well developed and overlapping intestine ventrally. Hemizonid often just above SE-pore, but sometimes seen at same level of SE-pore. Rectum usually inflated. Tail gradually tapering to a distinct hyaline terminus, clearly delimitated anteriorly, and a finely rounded terminus. Hyaline part about a quarter of tail length, with usually one or two constrictions.

#### Females

Body large with often well-pronounced elongated neck, average length of 0.8 mm with neck and width of 0.4 mm, clear annulation at neck region, pearly white color and globular in shape. Stylet thin, 16–20 μm, conus tip slightly curved dorsally; knobs rounded to transversely ovoid, slightly sloping backward. SE-pore between stylet knobs and metacorpus level. Perineal pattern rounded to oval, striae course, dorsal arch relatively low, lateral lines weakly visible.

#### Males

Body large, 1.0 to 1.8 mm long, tapering anteriorly. Lateral field with four lines forming three bands of equal width; outer bands with irregular but mostly complete areolations and mid band with sparsely scattered, incomplete areolations. Head continuous with body, or sometimes appearing slightly set-off, rounded, with single post-labial annule. Labial disc curved, elevated and fused with medial lips. Head region with incomplete transverse striations. Stylet robust, 22 to 25 μm long; knobs transversely ovoid and slightly backwardly sloping from shaft. DGO at 2.8 to 3.7 μm from knobs. Hemizonid 4 to 5 annuli anterior to SE-pore. Duct near SE-pore well sclerotized. Pharyngeal lumen usually well visible until valves of median bulb, pharyngeal gland lobe overlapping intestine ventrally. Tail short and twisted. Spicules 30 to 39 μm long. Gubernaculum simple, slightly curved ventrally.

### Remarks

The current population is morphologically similar to the original description of *M. paranaensis* (Carneiro et al., 1996) and also to the populations from Brazil and Guatemala (Santos et al., 2020). However, in these compared populations, a distinct sclerotization of the duct near the SE-pore of male was not mentioned, whereas such a sclerotization was prominent in all the males of our population. Therefore, this characteristic should be re-examined in the type specimens, and also in the Guatemalan population of *M. paranaensis* mentioned above, to ensure that it is a characteristic that separates it from other very similar species, such as *M. incognita*. Furthermore, the esterase phenotype of the herein-studied population was identical to that of the Guatemalan *M. paranaensis* population (see details under molecular characterization) (Santos et al., 2020). The exact location where the currently studied sample was collected in Guatemala remains unknown. Permanent mount of females, J2, and males have been deposited at the Wageningen Nematode Collection (WaNeCo), Wageningen, the Netherlands, and also at the UGent Nematode Collection of the Nematology Research Unit of Ghent University, Belgium.

### Molecular characterizations and phylogenetic analysis

Three identical sequences, each of the D2–D3 expansion segment of 28S (OQ772268–OQ772270; 735–740 bp), partial 18S (OQ772271–OQ772273; 860bp) and partial ITS (OQ772265–OQ772267; 709–713 bp) of rDNA, and five identical sequences of *cox*1 of mtDNA (OQ773540–OQ773544; 375–415 bp) were generated for *M. karsseni* n. sp. The D2–D3 sequences were found to be closest to an unidentified *Meloidogyne* sp. (JX465613; 99.6% identity; 3 out of 716 bp difference); the 18S sequences were closest to *M. incognita* (JX100421; 99.8% identity; 2 out of 854 bp difference); the ITS sequences were closest to *M. luci* Carneiro, Correa, Almeida, Gomes, Mohammad Deimi, Castagnone-Sereno and Karssen, 2014 (LN626964; 98.4% identity; 10 out of 641 bp difference); and the *cox*1 sequences was identical to *M. incognita* sequence (ON276646; 100% identity).

Similarly, for *M. paranaensis,* three D2–D3 of 28S (OQ753107–OQ753109; 630–730 bp long; 98.3–98.6% similarity; 14–15 bp differences), two identical partial 18S (OQ750410–OQ750411; 850 bp) and one partial ITS (OQ753116; 707 bp) sequences of rDNA, and three identical *cox*1 (OQ750575–OQ750577; 360–410 bp) of mtDNA were generated. The D2–D3 sequences were found to be closest to *M. incognita* sequence (MT193445; 99.6% identity; 4 out of 630 bp difference); the 18S sequences were closest to *M. arabicida* sequence (AY942625; 99.7% identity; 2 out of 850 bp difference); the ITS sequences were closest to *M. incognita* sequence (KP901064; 97.2% identity; 25 out of 709 bp difference); and the *cox*1 sequences were also closest to *M. incognita* sequence (ON276646; 99.5%; 2 out of 414 bp differences). Interestingly, upon comparison of the D2–D3 and the ITS sequences with the available corresponding sequences of *M. paranaensis* from Guatemala (Santos et al., 2020), our sequences were found to be relatively dissimilar to KY911103 (97.2–98.6% identity, 9 to 24 bp differences) and KY911109 (92.3% identity, 58 out of 730 bp difference).

In Santos et al. (2020), the 28S and the ITS sequences from the Brazilian and the Guatemalan *M. paranaensis* populations showed remarkable intraspecific sequence variations, up to 2%, and 8%, respectively. In the current study, a similar level of variation in the 28S (up to 3%) and the ITS sequences (7.3%) were also observed between our population and the Guatemalan population (Santos et al., 2020), indicating that these gene regions may not be reliable for the identification of *M. paranaensis*.

**Figure 7:**
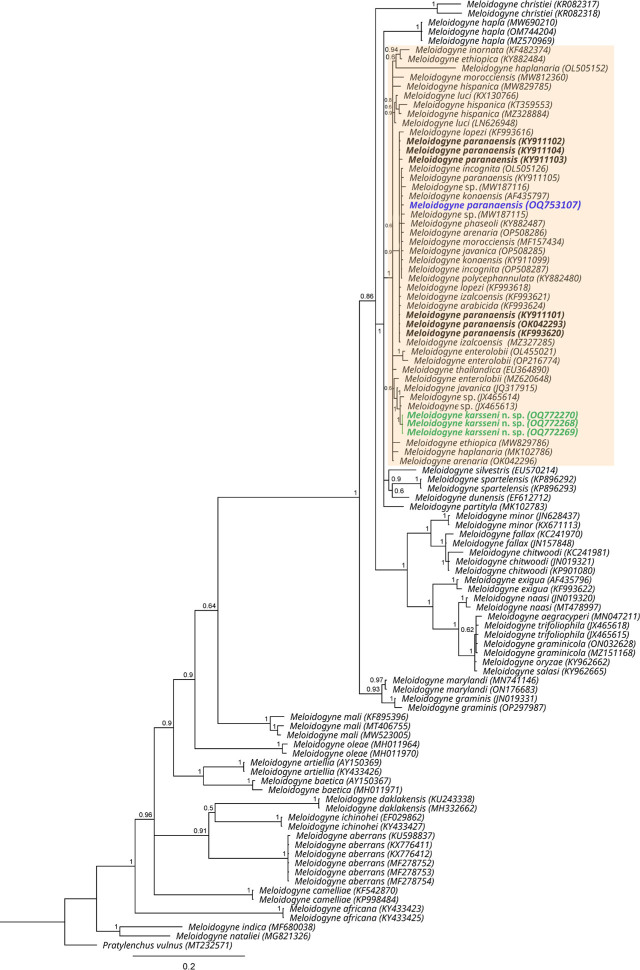
Phylogenetic relationships of *Meloidogyne karsseni* n. sp. (green) and *M. paranaensis* (blue) with other *Meloidogyne* spp. based on the Bayesian analysis of the D2–D3 of 28S rDNA sequence alignment using the GTR + I + G nucleotide substitution model. Posterior probabilities of more than 0.5 are given for appropriate clades. Tropical *Meloidogyne* species complex is highlighted in the colored box and the *M. paranaensis* sequences included in Santos et al. (2020) are in bold.

**Figure 8:**
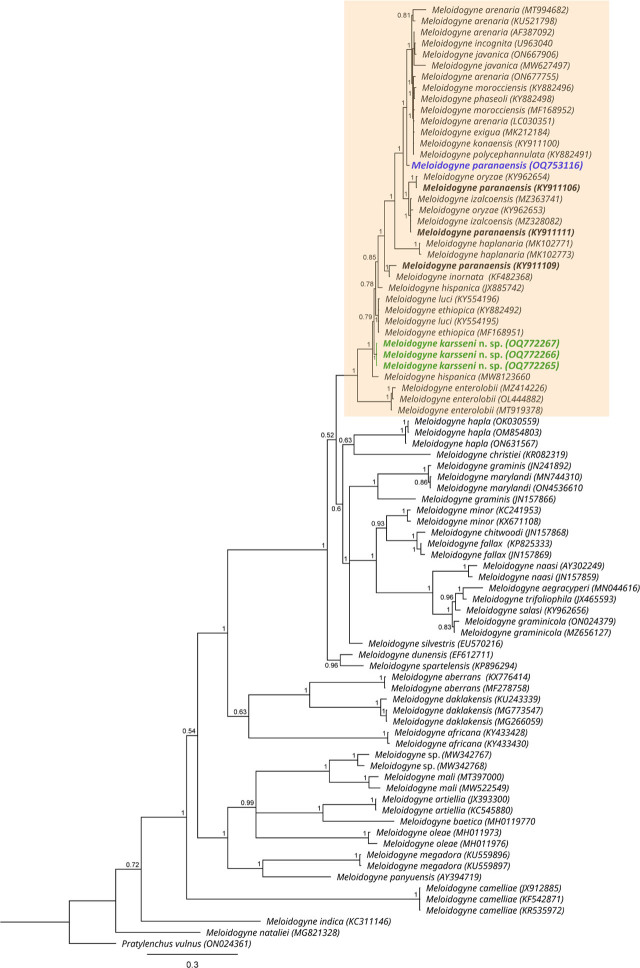
Phylogenetic relationships of *Meloidogyne karsseni* n. sp. (green) and *M. paranaensis* (blue) with other *Meloidogyne* spp. based on the Bayesian analysis of the ITS sequence alignment using the GTR + I + G nucleotide substitution model. Posterior probabilities of more than 0.5 are given for appropriate clades. Tropical *Meloidogyne* species complex are highlighted in colored box and the *M. paranaensis* sequences included in Santos et al. (2020) are in bold.

Based on the D2–D3 tree (770 bp-long alignment) (Fig. 7), both *M. karsseni* n. sp. and *M. paranaensis* were placed together in an unresolved clade (PP = 0.6) consisting of only tropical and subtropical RKN species (also known as Tropical RKN complex) (Álvarez-Ortega et al., 2019). Similarly, in the ITS tree (1000 bp-long alignment) (Fig. 8), both species appear in the tropical and subtropical RKN species clade (PP = 1), but the phylogenetic position of *M. karsseni* is only slightly resolved, i.e., it is sister to 16 species (PP = 0.79) (see trees for the respective related species). As 18S and *cox*1 tropical and subtropical RKN sequences were also highly conserved, and thus yielded similar tree topologies with an unresolved clade of tropical and subtropical RKN species, they are not shown here.

**Figure 9:**
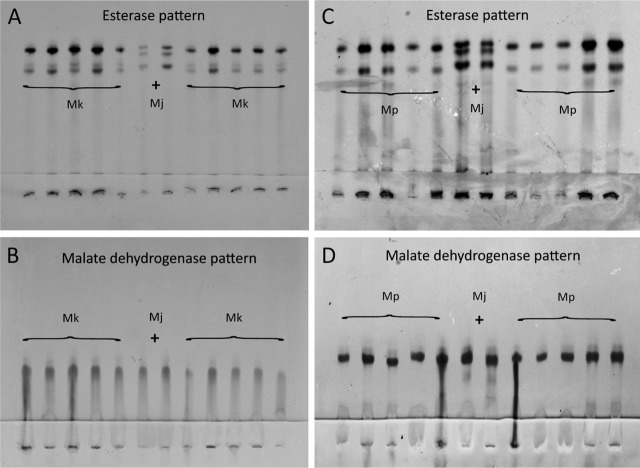
Isozyme characterizations of *Meloidogyne karsseni* n. sp. (*Mk*) and *M. paranaensis* (*Mp*) young females. A: Esterase pattern, B: Malate dehydrogenase pattern of *M. karsseni* n. sp., C: Esterase pattern, D: Malate dehydrogenase pattern of *M. paranaensis*. *Meloidogyne javanica* (*Mj*) was used as a reference sample and is shown using a plus sign in the middle two positions of the gels.

The isozyme codes (Fig. 9) of esterase phenotypes and malate dehydrogenase phenotypes of *M. karsseni* n. sp. are, respectively, F1-I1-S1 and N1 types. For *M. paranaensis*, they are F1–S1 (= P2a) and N1, respectively. The isozyme codes for the new species have not previously been seen, whereas those of *M. paranaensis* were identical to the corresponding codes of *M. paranaensis* from Guatemala (Santos et al., 2020).

## Discussion

A review by Subbotin et al. (2021) revealed the presence of 13 RKN species in Mexico and the Central American countries of Costa Rica, El Salvador, Guatemala, Honduras, Nicaragua, and Panama. These species include *M. arabicida*, *M. chitwoodi* Golden, O’Bannon, Santo & Finley, 1980, *M. cruciani* García-Martinez, Taylor & Smart, 1982, *M. enterolobii* Yang & Eisenback, 1983, *M. exigua* Göldi, 1887, *M. hispanica* Hirschmann, 1986, *M. incognita*, *M. izalcoensis*, *M. lopezi*, *M. luci*, *M. marylandi* Jepson & Golden, Jepson, 1987, *M. paranaensis* and *M. salasi* López, 1984. This number is expected to increase in the future as additional RKN samplings take place in these regions.

Many of these species are highly polyphagous and parasitize a wide range of economically important crops, whereas only a select few of them have been reported as parasitizing specific hosts. For example, *M. enterolobii*, *M. exigua, M. incognita* and *M. luci* have multiple hosts, ranging from vegetables such as beans (several plants in the family Fabaceae), tomatoes, tubers and other cash crops such as coffee (*Coffea arabica* L.), tobacco (*Nicotiana tabacum* L.), and cotton (*Gossypium hirsutum* L.) (Orton Williams, 1973; Oliveira et al., 2005; EPPO, 2014).

In contrast, RKN species such as *M. arabicida* and *M. lopezi* have only been reported in coffee (López & Salazar, 1989; Bertrand et al., 2000; Villain et al., 2013; Humphreys-Pereira et al., 2014). Interestingly, most of the above species (except for those with very specific hosts) have been found to parasitize on tomato (Sikora et al., 2018; Subbotin et al., 2021), also including the two species in this manuscript. The herein studied *M. paranaensis* population was originally found in tomato in Guatemala, although its primary host is coffee, and it has also displayed an ability to induce severe galling on the root system of tomato. Furthermore, even if the host range of *M. karsseni* n. sp. remains to be fully elucidated, it is known to thrive on tomato despite the fact that it was originally found in sweet pepper.

The morphological separation of the tropical RKN species complex has always represented a difficult challenge, even for nematode taxonomists. Traditionally, researchers have relied on morphometrics and morphology of perineal patterns for species identification, an approach known to be greatly hampered by phenotypic plasticity and interspecific similarities (Hunt & Handoo, 2009; Janssen et al., 2016). Nevertheless, *M. karsseni* n. sp. described herein can be morphologically and biochemically separated from all other known RKN species. The most important morphological characters that separate this new species from all the others include the DGO lengths and the female perineal pattern (i.e., rounded-to-oval perineal patterns with coarse striations, especially on the lateral sides around the anus, and low dorsal arch with fine striations). Conversely, molecular separation of both *M. karsseni* n. sp. and *M. paranaensis,* based on genetic markers (i.e., rRNA genes and *cox*1), was not definitive, because the sequences were highly conserved (see molecular characterization above). Furthermore, this resulted in poor resolutions of the phylogenetic trees, with many tropical and subtropical RKN species remaining unresolved, which is in agreement with similar findings in previous studies (Janssen et al., 2016; Álvarez-Ortega et al., 2019).

It is hopeful, however, that newer molecular markers, such as *nad*5 and intergenic COII-16S gene region of mtDNA, can reveal informative mitochondrial haplotypes. These markers have been shown to be more consistent and better-linked with the traditional esterase isozyme patterns (Janssen et al., 2016; Subbotin & Burbridge, 2021). The generation of these newer markers for *M. karsseni* n. sp. should be carried out in due course.
